# A framework for nosocomial transmission of emerging coronaviruses

**DOI:** 10.1017/ice.2020.296

**Published:** 2020-06-11

**Authors:** Seth D. Judson, Vincent J. Munster

**Affiliations:** 1 Department of Medicine, University of Washington, Seattle, Washington; 2 Laboratory of Virology, Division of Intramural Research, National Institute of Allergy and Infectious Diseases, National Institutes of Health, Hamilton, Montana

*To the Editor—*Over the past 17 years, 3 coronaviruses have emerged and caused diseases with high case fatality rates. From the severe acute respiratory syndrome (SARS) epidemic of 2002–2003, to outbreaks of Middle East respiratory syndrome (MERS) since 2013, to the pandemic of coronavirus disease 2019 (COVID-19), coronavirus diseases have afflicted global communities. Nosocomial, or healthcare-associated infections, have been recognized with each of these diseases because the viruses that cause these diseases are contagious, are relatively stable on surfaces, and are potentially disseminated through medical procedures. With the emergence of severe acute respiratory syndrome coronavirus 2 (SARS-CoV-2), the virus that causes COVID-19, many have wondered whether personal protective equipment (PPE) and hospital protocols are adequate to prevent transmission. To answer these questions, it is helpful to examine prior data for severe acute respiratory syndrome coronavirus 1 (SARS-CoV-1) and Middle East respiratory syndrome coronavirus (MERS-CoV).

Transmission of a virus occurs when an individual sheds viable virus that infects a susceptible host either through direct contact, through indirect contact with a contaminated surface (fomite transmission), or by exposure to virus-laden particles suspended in air. These particles are aerosols, which are often divided by size into large and small droplets.^1^ The term droplet transmission refers to infection via large droplets, and airborne transmission refers to small droplets. Aerosol transmission can generally refer to both categories of particles.^1^ These terms and the exact cut-off for droplet size are controversial. Given these different routes of transmission, research regarding nosocomial transmission of emerging viruses should address the following questions: Where is viable virus shed from infected individuals? How stable is the virus on surfaces, in liquids, and within aerosols in clinical settings? Through what routes of exposure and dosages of virus does infection occur? And lastly, in what situations are nosocomial transmission events occurring? Using this framework, we can assess the risks for nosocomial transmission of emerging coronaviruses. These characteristics for SARS-CoV-1, MERS-CoV, and SARS-CoV-2 are shown in Table 1.

**Table 1.** Characteristics of Emerging Coronaviruses and Nosocomial Transmission

Virus	Location of Shedding	Stability	Receptor	Cases Among HCWs, No (%)	Associated Settings/Procedures
SARS-CoV-1	URT^3^ LRT^3^ Urine^3^ Feces^3^	Similarly stable in aerosol as SARS-CoV-2^9^	ACE-2	10,002 (18.8) China 2003^2^ 386 (22) Hong Kong 2003^2^ 97 (40.8) Singapore 2003^2^ 36 (57.1) Vietnam 2003^2^ 109 (43.4) Canada 2003^2^	Settings: hospital Procedures: CPR, bronchoscopy, noninvasive ventilation, intubation, manual ventilation^1^
MERS-CoV	URT^5^ LRT^5^ Urine^5,a^ Feces^5,a^	More stable on surfaces at temperate than tropical conditions^4^ Decreased stability in aerosol with increased relative humidity^4^	DPP4	106 (13.5) Saudi Arabia 2013–2015^2^ 25 (13.4) South Korea 2015^2^	Settings: hospital, dialysis unit
SARS-CoV-2	URT^8^ LRT^8^ Feces^8^	Similarly stable in aerosol as SARS-CoV-1^9^ Detected on hospital surfaces and in aerosol samples^10,a^	ACE-2	1,716 (3.8) China 2020^6^ 2,026 (9) Italy 2020^7^	Settings: hospital, nursing facility

Note. HCW, healthcare worker; URT, upper respiratory tract; URT, lower respiratory tract.

a RNA detected, not confirmed viable virus.

During the initial epidemic of SARS, many healthcare workers (HCWs) were infected, with estimates ranging from 18.8% to 57.7% of the total cases within outbreaks.^2^ Retrospective studies showed that SARS-CoV-1 transmission was associated with certain aerosol-generating medical procedures (AGMPs), which can either generate or induce a patient to form virus-laden aerosols.^1^ For SARS-CoV-1 transmission, these included cardiopulmonary resuscitation, bronchoscopy, noninvasive ventilation, intubation, and manual ventilation.^1^ Viable SARS-CoV-1 was found to be shed via secretions in the upper and lower respiratory tracts (URT and LRT), urine, as well as in feces from patients.^3^ The angiotensin-converting enzyme 2 receptor was identified as the entry point for the virus to infect cells in the respiratory tract. Therefore, it was presumed that direct and indirect contact were likely sources of transmission. Given the association with AGMPs and detection of virus in the LRT and URT, aerosol transmission was also likely, although the specific relationship of aerosol size with infection was unclear.

When MERS-CoV emerged in 2013, healthcare settings were recognized as areas of outbreak amplification and possible super-spreading events.^2^ Multiple cases of MERS among HCWs were linked to hospital facilities in Saudi Arabia and South Korea.^2^ Experimental studies of MERS-CoV found that the virus was more stable on surfaces in temperate versus tropical environmental conditions and that the stability of the virus in aerosol decreased with increasing relative humidity.^4^ These findings indicated that healthcare environments could be particular areas of virus persistence. MERS-CoV was detected in bodily fluids, similar to SARS-CoV-1, but MERS-CoV utilized a different host cell receptor for entry, dipeptidyl peptidase 4 (DPP4), and it predominantly replicated in the LRT, indicating potential differences in transmission.^5^

As reports emerged about a disease caused by a novel coronavirus in China, which became known as COVID-19 and SARS-CoV-2, respectively, nosocomial transmission was again suspected. During the initial outbreak in China, 1,716 COVID-19 cases were confirmed (3,019 suspected) among HCWs as of February 11, 2020, and some of these infections likely occurred in healthcare settings.^6^ Subsequently, the pandemic spread to Italy, where at least 2,026 HCWs had been confirmed to have COVID-19 as of March 15, 2020.^7^ As the United States became a new epicenter of the pandemic, additional infections among HCWs occurred. Similar to SARS-CoV-1, viable SARS-CoV-2 was identified in the URT, LRT, and feces of patients, and SARS-CoV-2 was also found to use the ACE-2 receptor.^8^ Stability studies found that the virus was similarly stable to SARS-CoV-1 on surfaces and in aerosols.^9^ Multiple hospital surfaces and air samples were also found to be contaminated with SARS-CoV-2 RNA.^10^ Meanwhile, ongoing studies are evaluating where viable virus can be detected in clinical settings and whether certain medical procedures are associated with transmission. During these studies, it will be important to understand the variety of environments in different healthcare facilities. Given the similar stability of SARS-CoV-1 and SARS-CoV-2, AGMPs likely pose an increased risk for aerosol transmission of SARS-CoV-2, and healthcare surfaces could be sources of fomite transmission. As the pandemic continues to unfurl, it will be critical to identify which HCWs and patients may have been infected in clinical settings and through which route of transmission. Such research will not only allow healthcare systems to improve policies regarding PPE and decontamination procedures but will also enable risk assessment for healthcare personnel and patients. Although experiments can help us understand characteristics of emerging viruses, ultimately, multidisciplinary collaborations are required in clinical settings to elucidate and prevent nosocomial transmission.
